#  Cardiotoxicity of Molecularly Targeted Agents

**DOI:** 10.2174/157340311799960636

**Published:** 2011-11

**Authors:** Nadia Hedhli, Kerry S Russell

**Affiliations:** 1 Department of Internal Medicine-Section of Cardiovascular Medicine, Yale School of Medicine, New Haven, Connecticut, USA

**Keywords:** Cardiac toxicity, chemotherapy, cardiomyopathy, tyrosine kinases, proteasome inhibitors.

## Abstract

Cardiac toxicity of molecularly targeted cancer agents is increasingly recognized as a significant side effect of chemotherapy. These new potent therapies may not only affect the survival of cancer cells, but have the potential to adversely impact normal cardiac and vascular function. Unraveling the mechanisms by which these therapies affect the heart and vasculature is crucial for improving drug design and finding alternative therapies to protect patients predisposed to cardiovascular disease. In this review, we summarize the classification and side effects of currently approved molecularly targeted chemotherapeutics.

## INTRODUCTION

The explosion of novel therapies with specific cellular targets for treatment of cancer has brought both tremendous oncologic success and worrisome unanticipated cardiovascular toxicities. In this article, we will review the myriad of cellular pathways being targeted in cancer cells and why altering these pathways in cardiovascular cells has been shown, or may be predicted, to interfere with normal cardiovascular function. Involvement of cardiologists in development and clinical use of molecularly targeted chemotherapeutics is critical to allow patients to survive their cancer without irreversibly damaging the heart and vasculature. 

## MOLECULAR TARGETS: CLASSIFICATIONS, CURRENTLY APPROVED DRUGS, KNOWN AND ANTICIPATED SIDE EFFECTS

The term “molecularly targeted” broadly refers to therapeutic agents that have been designed to alter cellular pathways shown to be critical for cancer cell survival or proliferation Table **I**. These agents can either interfere with pathways that promote survival or growth (e.g. tyrosine kinase inhibitors (TKi)) or augment pathways that promote differentiation (e.g. retinoids). The type of damage that these agents cause to the heart has been grouped into Type I and Type II injury [1]. Type I injury is best exemplified by the cardiac effects of anthracyclines (reviewed in Chapter 1 of this series). Cardiac dysfunction (most commonly declines in systolic function) seen in response to this type of injury can be seen from the earliest administration of drug, is associated with histologically-detectable alterations in myocyte structure (including myocyte cell death) is dose-related, and is often irreversible. Type II injury was first described based on the observed effects of trastuzumab (discussed below). This toxicity is less predictably associated with dose, not generally associated with gross histological changes or myocyte death, and is often reversible (though long term effects remain to be fully evaluated and understood for many of these new drugs) [2].

In some cases, clinical cardiotoxicity resulting from targeting of these cellular pathways has lead to novel discoveries of their importance in preservation of cardiac structure and function. One example of this is the anti-erbB2 targeted agent, trastuzumab (Herceptin). We will begin our overview of this field by discussing the cardiotoxicity of this class of drugs.

### Growth Factor Signaling Pathways

#### ErbB (Epidermal Growth Factor) Family Receptors and the Trastuzumab Story

##### ErbB Receptors, Cancer and the Heart

The erbB family of receptor tyrosine kinases was recognized to participate in cellular proliferation and survival over 30 years ago [3-5]. The discovery that erbB2 (also called Her2 or neu) plays a critical role in some types of human breast cancer [6] rapidly led to strategies aimed at interrupting the signaling function of this receptor. Blocking erbB2 signaling in cancer cells was shown to stop cellular proliferation and increase susceptibility to agents promoting cell death. The first anti-erbB2 agent developed was trastuzumab, a humanized monoclonal antibody directed at the extracellular ligand-binding domain of erbB2. Early trials demonstrated the efficacy of trastuzumab in patients with the most severe and resistant forms of metastatic breast cancer [7]; however, excitement over this novel therapy was soon tempered by the observation that as many as 25% of patients receiving this therapy went on to develop declines in cardiac systolic function [8]. It was rapidly recognized that both cardiac myocytes [9] and cardiovascular endothelium [10] express erbB receptors, and that interruption of erbB signaling pathways results in both cardiac developmental defects [11] and increased susceptibility of cardiac myocytes to injury in animal models [12, 13]. 

##### Potential Mechanisms of Anti-erbB-targeted Cardiotoxicity

Though both cardiac myocytes and endothelium express erbB receptors, the main focus of attention regarding the target of cardiotoxicity in the heart has been the myocyte. Several mechanisms have been proposed to explain the putative toxic effects of trastuzumab on the adult cardiac myocyte based on the effects of activating erbB signaling using the ligand, neuregulin. Neuregulin-mediated erbB receptor activation has been shown to activate MER/ERK signaling and increase hypertrophic gene expression and protein synthesis [14]. ErbB mediated activation of ERK has also been shown to regulate sarcomere synthesis/organization and stability [15]. Another signaling pathway induced by erbBs in myocytes is the PI3-kinase/Akt pathway. AKT is well known to be important in protection of myocytes against cell death and also a key regulator of cell metabolism and growth. Thus, trastuzumab has the potential to interrupt erbB-mediated cell protection/survival, cytoskeletal architecture, EC (excitation/contraction) coupling, and metabolism. Based on the absence of significant histological changes in hearts from patients treated with trastuzumab and the frequent reversibility of cardiac dysfunction in patients treated with this drug [16] the trastuzumab-induced cardiotoxicity has been classified as Type II. These findings make it unlikely that the mechanism of cardiotoxicity is significant, irreversible myocyte damage or cell death. 

Though the myocyte is clearly a potential significant target for trastuzumab, it is also important to note that erbB receptors and their ligand, neuregulin, are also expressed by vascular and endocardial endothelial cells. Thus, endothelium is also an important potential target for anti-erbB-targeted therapies [17]. The activation of endothelial erbB receptors in response to neuregulin release in endothelial cells has been shown to promote angiogenesis *in vivo *[10], and increase vascular endothelial growth factor (VEGF) expression in tumor cells [18]. Loss of this signaling may result in impaired endothelial proliferation and survival. Many studies support the important critical role of cardiac endothelial cells in autocrine/paracrine regulation of the heart. Removal of endocardial or vascular endothelium has been shown to result in a decline in myocyte contractile function [19]. Furthermore, loss of coronary microvasculature has been shown to be an important contributor to the pathophysiology of many common forms of cardiovascular disease, including hypertension and diabetes [20]. Endothelial-specific deletion of neuregulin has been shown to increase myocyte susceptibility to ischemia-reperfusion injury, and exogenous administration of neuregulin can protect the heart from this injury [13]. Thus, interruption of this protective pathway by anti-erbB-targeted therapies may result in cardiotoxicity both due to myocyte and endothelial damage. 

##### Clinical Cardiotoxicity of Anti-erbB2 Targeted Therapies: Assessment, Prevention and Treatment 

The rate of clinically significant cardiac events (declines in left ventricular ejection fraction (LVEF), including congestive heart failure (CHF)) have been reported in 0.5-19% of patients in the HERA and NSABP trials of trastuzumab [21, 22]. Based on observations from multiple clinical trials, the manufacturer (Genentech) has placed specific guidelines for monitoring for cardiotoxicity in patients receiving trastuzumab [23]. LVEF should be measured at baseline (prior to starting therapy), at 3-month intervals during therapy, and at 6-month intervals for at least 2 years following completion of therapy. The drug should be held for 4-8 weeks if: 1) there more than a 15% drop in LVEF from baseline, but EF remains above the lower limit of normal; or, 2) there is more than a 10% drop from baseline to a level that is below the lower limit of normal. The drug may be restarted after the hold period if repeat imaging demonstrates LVEF is above the lower limit of normal and is not >15% below the baseline LVEF. Trastuzumab should be discontinued permanently if the patient develops CHF, has a significant asymptomatic decline in LVEF (as above) that persists for >8 weeks, or has had the drug held on more than 3 occasions due to declines in LVEF [23]. Either echocardiography or MUGA are acceptable imaging modalities for this assessment. Though the majority (79% [24]) of patients who experience declines in LVEF recover in the short-intermediate term, the long term consequences of LV dysfunction in these patients remains to be seen [2, 25]. Administration of standard heart failure medications, including angiotensin converting enzyme inhibitors (ACEi) and carvedilol, has been advocated for all patients with significant declines in LV function and has been associated (though not in any randomized trials) with recovery of LV function [26]. Recent data suggests that patients who have a positive troponin before or during trastuzumab therapy have a higher incidence of LV dysfunction and a lower likelihood of recovery, suggesting that additional biomarkers may help in prognostication of these patients [26]. The finding of positive troponins in these patients also suggests that in the most severe cases of trastuzumab-induced injury, myocyte necrosis (either as a direct effect on cardiomyocytes or as an indirect effect of vascular toxicity) can occur and lead to irreversible damage. Additional risk factors for trastuzumab-induced cardiotoxicity include age > 60 years and prior or concomitant treatment with anthracyclines [24]. Whether other traditional cardiac risk factors play a role in risk of trastuzumab-induced cardiotoxicity is unclear at this point, though treatment of underlying risk factors (e.g. hypertension) has been advocated [27]. Patients at higher risk of developing chemotherapy induced cardiac dysfunction may benefit from cardioprotection using ACEi (or carvedilol), as has been shown in small clinical trials of patients undergoing anthracycline-based chemotherapy [28, 29]. However, clinical data to support this idea is currently lacking. Despite this, the United Kingdom National Cancer Research Institute (NCRI) has made revisions to its recommendations that include starting ACEi and referral to a cardiologist for all patients with baseline cardiac dysfunction or those whose LVEF shows a significant decline while on trastuzumab [27]. Future studies of additional biomarkers (e.g. serum natriuretic peptides or neuregulin) and of efficacy of ACEi or carvedilol in prevention or treatment of trastuzumab-induced cardiac dysfunction are underway and should provide additional information in this patient population. Interestingly, despite similar targeting to interrupt erbB2 signaling, the small molecule kinase inhibitor lapatinib has shown significantly less cardiotoxicity. In a meta-analysis of 43 clinical trials including over 3500 patients, the incidence of declines in LVEF was 1.6% with only 0.2% of patients experiencing symptomatic CHF [30, 31]. This observation emphasizes the need to study each individual targeted drug separately for potential cardiac side effects.

#### VEGF (Vascular Endothelial Growth Factor) and its Receptors: Targeting the Blood Supply of Cancer

##### Rationale for Anti-VEGF Targeted Chemotherapy 

The essential role of angiogenesis in tumor progression was first proposed by Folkman in 1971 [32]. VEGFA was found to be the central proangiogenic protein that is expressed and secreted by more than 60% of human cancers [33, 34]. VEGF is thought to promote tumor angiogenesis by acting on its cognate receptor VEGFR2 on endothelial cells to activate proliferation and survival pathways. Therapies targeted at interrupting this important pathway were believed to represent a crucial breakthrough in cancer therapy. Bevacizumab, a monoclonal antibody directed against VEGF, has been successfully used in a number of resistant cancers and was approved for use after successful trials in colorectal cancer in 2004 [35]. Interestingly, interruption of VEGF signaling may not primarily act through impaired angiogenesis to mediate effects of these drugs on tumorigenesis, but may also “normalize” leaky tumor vasculature to allow more efficient delivery of additional chemotherapeutic drugs and by altering endothelial progenitor cell homing and function in the vasculature [36, 37].

##### Clinical Cardiovascular Toxicity of Anti-VEGF Therapies: Hypertension

Because vascular endothelium is a major target of this therapy, side effects (on target) on normal vasculature might be anticipated. The most frequent cardiovascular side effect of this class of drugs is hypertension, which has occurred in 30-80% of patients undergoing clinical trials with anti-angiogenic drugs [38, 39]. This hypertension appears to be dose-related [40], and may occur rapidly (as early as 24 hours with some VEGF inhibitors) [41]. The risk factors for developing severe hypertension are still somewhat unclear, but may include increased age (>65 years), smoking, hypercholesterolemia, and prior hypertension [38, 42]. The mechanism for hypertension is not completely understood, although VEGF is known to lower blood pressure (likely through a nitric oxide-dependent mechanism) [43]. Secondary causes of hypertension, such as activation of the renin-angiotensin pathway, do not appear to play a significant role in this process, though vascular stiffness appears to increase [44]. Similarities to hypertension seen during preeclampsia, where VEGF signaling has also been shown to be decreased by overproduction of soluble VEGFR-1 (sFlt1), have led to speculation that hypertension (and proteinuria) seen with anti-VEGF therapy may also be due to decreased VEGFR-2 signaling in EC with resultant decreased NO production, vasoconstriction and hypertension [45]. The severity of anti-VEGF associated hypertension can range from mild to extreme, including hypertensive urgency and crisis. Thus, treatment of anti-VEGF-induced hypertension is clinically important. Because hypertensive responses to anti-VEGF therapy have been suggested to correlate with tumor response [46], some patients may be hesitant to receive treatment for this hypertension. No specific recommendations for treatment of anti-VEGF-induced hypertension are currently available, thus, treatment has usually followed the existing guidelines for anti-hypertensive treatment [47]. This should include counseling on dietary and lifestyle modification, avoidance of additional drugs that may exacerbate hypertension, and tailored therapy depending on comorbid diseases and other individual patient factors. 

Besides hypertension, there are several additional cardiovascular side effects seen with bevacizumab that overlap with those seen for other anti-angiogenic (including anti-VEGF) therapies, these will be discussed below.

#### Multi-targeted Kinase Inhibitors

##### Anti-angiogenic Targeted Inhibitors

Several small molecule inhibitors of VEGF signaling also target additional portions of angiogenic signaling pathways. Approved members of this class of drugs include sorafenib, pazopanib, and sunitinib and aflibercept. The signaling pathways affected by and reported side effects of these drugs are summarized in Table **I**. (It should be noted that for many of the newer molecularly targeted agent, no large scale dedicated trial data exists on specific cardiovascular effects. Thus, reported side effects may represent a single case or only a small number of patients). Though some of these anti-angiogenic targets are predominantly expressed on vascular endothelium, others are also expressed on cardiac myocytes and fibroblasts, thus the potential side effect profile in the cardiovascular system may be quite broad. All of these can also cause hypertension, probably also by virtue of their anti-VEGF effects [41, 44, 45, 48, 49], though additional effects such as elevating endothelin-1 levels, have also been described [50]. Next, we will briefly summarize some of the additional cardiovascular toxicities that have been described for these drugs and their potential mechanism(s) and treatment.

##### Thrombosis, Thromboembolism and Bleeding

Inhibition of VEGF signaling has been associated with endothelial dysfunction that may predispose to either thrombosis or bleeding [51]. Early trials of anti-angiogenic drugs appeared to show evidence of increased arterial thromboembolism, even causing myocardial ischemia, proposed to be an effect of endothelial cell activation [52]. Additionally, anti-VEGF therapy may also contribute to declines in platelet numbers or function as well as perturbations in coagulation cascades [53, 54]. The idea that patients receiving these therapies may have increased risk of either pro- or anti-thrombotic events has been suggested by data from several clinical trials. Two large trials of bevacizumab have shown bleeding and thromoboembolism in 1-3% of treated patients [55, 56]. Both venous and arterial thromboembolism have been described, with venous thromboembolism seen in 12-20% of patients [52, 57]. In a meta-analysis of clinical trials of bevacizumab, a 3.3% incidence of arterial thromboembolic events was seen, with relative risk of cardiac ischemia of 2.14 compared to controls (p=0.021). A meta-analysis of data regarding sunitinib and sorafenib have suggested that these drugs may be associated with increased relative risk of throboembolism up to 3 times that of control patients [58]. These findings raise the question of whether higher risk patients should receive aspirin or other anti-thrombotic pharmacotherapy. However, increased risk of bleeding with anti-angiogenic therapies (sorafenib and sunitinib) has also been suggested by some clinical data with a relative risk of 2 vs. controls, and an incidence of bleeding of 16% [59]. (Though, both these meta-analyses of thrombosis and bleeding have been criticized for failure to accurately estimate risk/unit time compared to controls [60, 61].) The incidence of bleeding with bevacizumab, in particular, may be higher. In a meta-analysis, the overall incidence of bleeding was 30% (high grade bleeding incidence 3.5%) with a relative risk of 2.48 [62]. Overall, increased awareness and monitoring for pro-thrombotic or bleeding events is certainly warranted, and is particularly relevant for many patients being seen by cardiologists in this setting. Clinical trials of patients receiving these medications in combination with aspirin or other anti-thrombotic medications will be required before recommendations can be made regarding risk or benefit of such interventions.

##### QT Prolongation 

Bevacizumab, sunitinib, and pazopanib have all been reported to cause prolongation of the QT interval [63]. This interaction can be exacerbated by co-administration of other QT prolonging drugs, therefore attention to these effects by monitoring serial ECGs is warranted. Baseline ECGs should be obtained in patients preparing to receive these medications, and QTc >450 msec for men or >470 msec for women should be used as cutoff for increased attention to monitoring during therapy. An increase in QTc of >60 msec over baseline or to a value >500 msec may place patients at significantly increased risk of arrhythmia [64, 65]. 

##### Angiogenesis Inhibitors and Left Ventricular Dysfunction

In experimental models, it has been shown that VEGF is up regulated after myocardial infarction or hypertrophy in response to increased afterload [66, 67]. Furthermore, blockade of VEGF signaling promotes progression from hypertrophy to dilated cardiomyopathy in the overloaded heart [68]. Taken together, these findings support the idea that interruption of VEGF signaling may lead to adverse cardiac remodeling and left ventricular dysfunction. Clinical trials of bevacizumab as adjunctive therapy for breast cancer have suggested that VEGF blockade may act synergistically with other types of chemotherapeutics with know cardiotoxicity [69]. A meta-analysis of phase I-III clinical trials of bevacizumab, sorafenib and sunitinib showed that 0.05-1.4% of patients had grade III-IV cardiac dysfunction (definitions available at http://ctep.cancer.gov/protocolDevelopment/electronic_applications/ctc.htm) after treatment with these drugs [70]. Sunitinib in particular has been shown to be associated with declines in LVEF in 7-28% of patients [71, 72]. Rates of clinically significant congestive heart failure range from 2.7-15% in various phase I-III clinical trials of sunitinib [71-74]. The risk of cardiac events in these patients was increased in those who developed hypertension (which occurred in 10-47% of patients) and those with underlying coronary artery disease or prior heart failure [71, 72]. Administration of sunitinib has been associated with mitochondrial and myocyte structural changes in animal models as well as in human biopsy specimens from patients who developed heart failure on this treatment [71, 75]. The reason for enhanced cardiotoxicity of sunitinib versus bevacizumab may be due to additional inhibition of platelet-derived growth factor (PDGF) signaling in addition to inhibition of VEGF blockade. Loss of PDGF signaling has been shown to result in cardiac dysfunction after load-induced stress, likely due to impaired paracrine angiogenic responses, in animal models [76]. This observation represent another excellent example of how unanticipated side effects of targeted therapies can lead to novel discoveries of the importance of these pathways in the cardiovascular system.

##### Other Multi-targeted Kinase Inhibitors

The use of Abl-targeted therapies (imatinib, dasatinib and nilotinib) has significantly improved survival for patients with chronic myelogenous leukemia (CML) [77]. Though initial reports suggested that inhibition of Abl kinase by imatinib leads to mitochondrial damage and cardiac myocyte cell death in both animal models and humans [78], more recent data suggests that this effect is only seen at very high doses that are not likely to be clinically relevant [79, 80]. In 3 clinical trials, no significant cardiac toxicity was seen with imatinib, even with up to 12 months of therapy and over 3 years of follow up [81-83]. However, the manufacturer reported retrospective data suggesting a rate of 0.2% of cardiotoxicity [84], thus it is still recommended that patients undergo cardiac monitoring (especially of LV function) during and after imatinib therapy [85]. Additional side effects of these drugs include QT prolongation (with dasatinib and nilotinib) and fluid retention (with imatinib and dasatinib) [86]. Though general fluid retention is a common side effect of imatinib therapy, pleural and pericardial effusions are less frequent (0-2%) [87]. Dasatinib therapy has been shown to be associated with pleural effusions much more frequently, in 14-43% of patients [88]. Up to 1/3 of patients with such pleural effusions also have pericardial effusions; however, these can usually be treated by reducing the dose of dasatinib, and using steroids and diuretics and very rarely require interventional drainage [88].

##### Intracellular Signaling Pathway Inhibitors: mTOR

The mammalian target of rapamycin (mTOR) emerged as a critical pathway driving tumor growth by promoting the synthesis of new blood vessels and over-expression of proteins that control the progression of cell through the cell cycle, cyclins. Thus, inhibitors of mTOR have emerged as a tool to control tumor growth [89]. The mTOR signaling axis acts *via* phosphorylating its two main downstream effectors, 4EBP-1 and S6K1, that regulate proteins synthesis. Rapamycin is a bacterial product that can inhibit mTOR by associating with its intracellular receptor FKBP-12. Similar to rapamycin, other small molecules such as temsirolimus (Torisel) bind to FKBP-12 to form a complex that interacts with the mammalian target of rapamycin kinase (mTOR), blocking its activity [90]. Temsirolimus is a potent and highly specific inhibitor of mTOR. It inhibits cell proliferation, cell growth, survival pathways and tumor angiogenesis [91]. The safety and effectiveness of temsirolimus were shown in a clinical trial of 626 patients divided into three groups: one received temsirolimus alone; the second, interferon alpha; and the third group received a combination of both drugs. The best median overall survival was observed in the group receiving temsirolimus alone [92]. The most common drug related adverse events associated with temsirolimus was anemia, however various adverse metabolic effects have also been reported, including elevations in serum glucose, triglycerides, and cholesterol [93]. In addition, interstitial pneumonitis has also been reported, and can be a life threatening toxicity associated with temsirolimus [94]. This novel inhibitor of mammalian target of rapamycin has been demonstrated to prolong overall survival and delay of disease in patients with advanced renal cell carcinoma [95]. The most frequent cardiovascular side effect reported with temsirolimus is hypertension [95]. Hypertension has also been reported with another inhibitor of mTOR, Everolimus (RAD-001). Similar to temsirolimus, Everolimus inhibits mTOR by binding to FKBP-12 and forming a complex that in turn binds and inhibits mTOR kinase, however this drug is administered orally unlike Temsirolimus, which is infused. In a phase III study of renal cancer, 410 patients that had failed a kinase inhibitor treatment were randomized into two groups: one that received placebo, and one that received Everolimus. The results of this trial showed an improved progression free survival when compared to placebo. This agent has been approved for usage in patients that have failed VEGF inhibitor treatment. Similar to the side effects observed with temsirolimus treatment, patients receiving Everolimus have reported to have skin rash, fatigue, hyperglycemia, and hyperlipidemia [96]. Furthermore, Everolimus has a potent immunosuppressive activity that was developed for the usage in acute and chronic rejection of solid organs. It inhibits smooth muscle proliferation and neointimal thickening and is used to reduce the risks of vasculopathy in heart transplantation [97]. 

As detailed previously mTOR may promote blood vessel formation, and blocking its signaling affects vessel growth, which may contribute to the hypertensive effects observed in patients. Dysregulation of mTOR signaling can lead to loss of expression of the von Hippel-Lindau tumor suppressor gene. This results in increased expression of hypoxia-inducible factor 1 (HIF-1) and its target gene products, such as VEGF. VEGF and other factors induced by HIF-1 are thought to be the key drivers of tumor angiogenesis [98]. Therefore, inhibition of mTOR may also affect VEGF expression, and have some of the same side effects described above for the anti-VEGF directed therapies [99].

##### Hormone Signaling Pathways

Some breast cancers are exquisitely sensitive to withdrawal of estrogen or inhibition of estrogen signaling through the estrogen receptor (ER). This had lead to very successful molecular targeting of ER and the aromatase enzyme responsible for the first steps of estrogen synthesis for treatment of these cancers. However, there are also multiple cell types in the cardiovascular system that contain ER and are hormone sensitive. The relationship between increased incidence of coronary artery disease and post-menopausal status as well as the failure of the hormone replacement clinical trials emphasizes the importance of estrogen signaling in the cardiovascular system [100]. Tamoxifen has mixed estrogenic and anti-estrogenic activities, and has both been associated with increased risk of thromboembolism including stroke [101]. This risk has been suggested to be lower with some other anti-estrogen therapies, despite a very similar mechanism of action, thus each drug may need to be evaluated individually for this effect [102]. Amongst patients with embolic phenomena on tamoxifen, there was an increased incidence of factor V Leiden mutations, and the presence of such mutations was associated with a 5-fold increase in risk of these events [103]. The thromboembolic risk of aromatase inhibitors has been shown to be less than or equivalent to that seen with tamoxifen [104-106]. 

Tamoxifen and toremifene have been shown to have favorable effects on lipid profiles, decreasing total and LDL cholesterol (both), and increasing HDL (toremifiene) [107, 108]. These effects may be predicted to be due to estrogenic effects of tamoxifen or toremifene, leading to the idea that aromatase inhibitors may have adverse effects on lipid profiles. Anastrozole and letrozole have been suggested to lead to mild increases in serum cholesterol; whereas, examestane appears to have cholesterol effects similar to tamoxifen and also may lower triglycerides, which may be due to its steroidal structure [109, 110]. There has been debate regarding risk of cardiovascular events in patients receiving aromatase therapy [111]. In the BIG 1-98 study, there was an excess of more severe adverse cardiac events, including ischemic heart disease and heart failure, in the letrozole-treated group compared with the tamoxifen group; however, the rates of hypertension and stroke were not significantly different, and the risk of thromboembolism was higher in the tamoxifen-treated group [112, 113]. Additional meta-analyses that included multiple aromatase inhibitors have also suggested a small, but significant increase in cardiovascular events on these drugs; however, whether these are a class effect or more attributable to one specific drug over another remains to be determined in additional trials [111, 114]

##### Regulators of Gene Expression, Cell Survival, and other Cellular Functions Histone Deacetylase Inhibitors

Histones are large proteins that bind to DNA to regulate its folding and accessibility for transcription. Acetylation of these proteins plays an important role in their regulation of gene expression by decreases binding of histones to DNA. This allows chromatin expansion and altered transcription of genes. This acetylation occurs in both normal hematopoietic cells and cancer cells allowing a wide range of transcription and expression of genes [115]. Two classes of enzymes can affect the acetylation of histones: histone acetyltransferases (HATs) and histone deacetylases (HDACs). Altered HAT or HDAC activity has been identified in several cancers, but HDAC inhibitors have been shown to be the most effective in inhibiting cancer growth [116]. HDACs play a critical role in modulating the balance between pro- and antiapoptotic proteins, and HDAC inhibitors activate apoptotic pathways [117]. In addition, HDAC inhibitors can inhibit angiogenesis by increasing acetylation of the proangiogenic factor HIF-1 alpha and enhancing its degradation [118] and decreasing the expression of VEGF receptor [119]. The HDAC inhibitor vorinostat (Zolinza) and romidepsin (Istodax) are approved for treating refractory cutaneous T-cell lymphoma [120] and other types of cancers. 

Although no major cardiotoxic events were reported with the usage of vorinostat, EKG changes and sudden cardiac death have been reported with romidepsin [121]. Tachycardia is the most common cardiovascular side effect reported with HDAC inhibitors. Adverse cardiac events have been reported with the HDAC inhibitor depsipeptide in patients with metastatic neuroendocrine tumors. In a study of 15 patients receiving depsipeptide palpitations, ventricular tachycardia (N=2) and sudden cardiac death (N=1), QTc prolongation (N=3), ST depression/ T-wave inversion, and T-wave flattening were reported [122]. Similar findings were seen in another study [123], leading to discontinuation of this drug as a therapeutic agent. Though the currently approved HDAC inhibitors have not had this degree of cardiotoxicity, monitoring may be warranted in selected high-risk patients.

#### Retinoic Acid Receptor Agonists

Retinoids are a family of signaling molecules structurally similar to vitamin A that act primarily by regulating gene expression and inducing cell differentiation [124]. Furthermore, retinoids have been reported to arrest cell cycle progression by modulating cyclins and cyclin-dependent kinases. Retinoid signaling is reported to decrease early in tumorigenesis suggesting that inhibition of this pathway is required for tumor growth. The main action of retinoids is through activation of the nuclear retinoic acid receptor therefore, these receptors using pharmacologic agonists. Currently 3 agonists, bexarotene (Targretin), alitretinoin (Panretin) and tretinoin (Vesanoid), have been clinically approved for the treatment of a wide range of tumors [125]. Though infrequent, cardiac toxicity has been reported with these treatments. For example, tretinoin treatment has been associated with pleural or pericardial effusions, hypotension and dysfunction in myocardial contractility [126-130].

#### Proteasome Inhibitors 

The ubiquitin proteasome system plays a central role in controlling the abundance of available proteins in the cells, and thus can stimulate and inhibit various pathways that plays central pathological and maintenance roles inside a cell. Proteins that are destined to be degraded by the proteasome are tagged by a ubiquitin chain in a process requiring the presence of specific ubiquitin ligases. Over activation of the ubiquitin-proteasome system has been documented in several types of cancers. In addition, the proteasome regulates the abundance of key transcription factors that promote tumor growth. For example, NFKB is well known to play a central role in driving tumor growth by promoting angiogenesis, cell invasion, proliferation and suppression of apoptosis. Proteasome inhibitors have been found to be efficient in regressing tumor growth. Bortezomib (Velcade) is an FDA approved proteasome inhibitor used for the treatment of patients with multiple myeloma. It efficiently regresses tumor growth by inducing apoptosis; however, this therapy has been associated with several case reports of congestive heart failure [131, 132]. Inhibition of proteasome activity affects a whole pool of different pathways, some of which are potentially required for survival of non-tumor cells, including cardiac myocytes. In addition, proteasome degradation is a central homeostasis pathway that is important during cardiac stress responses, and proteasome inhibition has been shown to interfere with compensatory cardiac hypertrophy and remodeling [133]. Cardiac side effects of proteasome inhibitors have been documented in clinical trials. In an extension study based on the SUMMIT and CREST trials, a case of cardiomegaly was reported [134]. In APEX, a phase III trial, 7 patients (2% of total patients studied) developed congestive heart failure [135]. Cases of severely decreased LVEF have also been reported [131].

## COPING WITH THE JANUS OF TARGETED THERAPIES

In summary, the explosion of information regarding the molecular mechanisms of carcinogenesis and tumor progression has lead to many new and promising therapeutics. However, many of the signaling pathways targeted for cancer therapy are also important for maintenance and repair in the cardiovascular system. Both predictable and unpredictable, on and off target effects may contribute to cardiotoxicity from these agents. Future goals of chemotherapeutic target design should benefit from advanced pre-clinical screening of cardiovascular target overlap to aid guiding therapeutics towards those pathways critical for interrupting cancer survival and progression that are non-essential for cardiovascular function. In addition, very little is known about identifying patients at risk for cardiotoxicity and whether traditional or novel drugs may aid in preventing cardiovascular side effects while preserving chemotherapeutic efficacy. Additional clinical trials may help identify such risk factors and protective strategies. Based on current data, limited recommendations for monitoring and treatment of patients receiving potentially cardiotoxic chemotherapeutics have been made primarily for those receiving anthracyclines and trastuzumab [27, 136]. In addition to these, it is critical for oncologists and cardiologists to familiarize themselves with all the potential cardiac side effects of these drugs, and in the absence of formal recommendations (which must await clinical trials), use their own judgment regarding the timing and use of cardiac monitoring. Specifically, we suggest that patients receiving any of the above discussed drugs with any data suggesting potential cardiotoxicity be considered for pre-treatment cardiac evaluation and more frequent monitoring if 1) they have underlying cardiovascular disease or dysfunction (including LV dysfunction, or baseline long QT, advanced age or other traditional cardiac risk factors), or 2) they are have previously received cardiotoxic chemotherapy or will be receiving concurrent therapy that is potentially cardiotoxic or will affect the QTc. Finally, in addition to the important role cardiologists can play in protecting the hearts of patients undergoing chemotherapy with these new and highly effective agents, we should be actively observing their effects with an eye towards developing novel therapies for patients with other types of cardiovascular diseases. 

## FUNDING SOURCES

This work was supported by R01HL80176 (KSR), K08HL04429 (KSR), and T32HL007950 (NH) from the NIH-NHLBI, and Grant in Aid (0355623T) from the American Heart Association (KSR). 

## Figures and Tables

**Table I.** 

Molecular Target	Drug	Type	Cancer target (approved)	Cardiovascular Targets (by Target) Cardiovascular Clinical Effects (by Drug) *
**Growth Factor Receptors and Intracellular Signaling Pathways**				
ErbB2 (Her2/neu)				Cardiacmyocyte, Vascular endothelium,
	Trastuzumab (Herceptin)	mAb	Breast, Gastric, GE Junction cancers	Declines (often reversible) in LV function.
	Lapatinib (Tykerb)	SMI	Breast cancers	Less frequent declines in LV function than trastuzumab, but reported. QT prolongation.
ErbB1 (EGFR)				Off target (other erbB or TKs) potential for both cardiac myocytes and endothelium
	Gefitinib (Iressa)	SMI	Non small cell lung cancer	No CV effects documented to date.
	Erlotinib (Tarceva)	SMI	NSCLC, pancreatic cancer	Thromboembolism (including stroke and MI)
	Cetuximab (Erbitux)	mAb	Head/neck squamous cell, colorectal carcinomas	Cardiopulmonary arrest, thromboembolism.[137]
	Panitumumab (Vectibix)	mAb	Metastatic colorectal cancer	Thromboembolism, edema
VEGF				Vascular endothelium
	Bevacizumab (Avastin)	mAb	NSCLC; metastatic colorectal, breast or kidney cancer; glioblastoma	Hypertension (including hypertensive crisis or hypertensive encephalopathy), hemorrhage (including CNS), thrombosis/embolism, congestive heart failure, myocardial infarction, stroke, syncope, pulmonary hypertension.
	Aflibercept (Zaltrap)	VEGF trap fusion protein	NSCLC; prostate, thymic, ovarian colorectal cancers	Hypertension, arrhythmia (most common supraventricular, bradycardia, ventricular extrasystoles). [138, 139]
**Multi-KinaseTargeted Drugs**				Cardiac myocyte (Abl), Vascular endothelium (PDGFR), Cardiac/Vascular progenitors (c-Kit)
VEGFR, PDGFR, Raf c-Kit, Flt3, RET	Sorafenib (Nexavar)	SMI	Renal cell, hepatocellular cancer	Hypertension, hemorrhage, congestive heart failure, cardiac ischemia, hypertensive crisis.[140]
VEGFR, PDGFR, FGFR, c-Kit, ltk, Lck, c-Fms	Pazopanib (Votrient)	SMI	Advanced renal cell cancer	Hypertension, chest pain, QT prolongation, thromboembolism (including myocardial infarction and stroke), hemorrhage
VEGFR, PDGFR, c-Kit, FMS, RET, Flt-3	Sunitinib (Sutent)	SMI	Metastatic renal cell, GI stromal cell	Declines in LV function, QT prolongation, hypertension, hemorrhage, peripheral edema, thromboembolism, thrombotic microangiopathy.[73]
Abl, c-Kit, PDGFR	Imatinib (Gleevec)	SMI	GI stromal tumor, some leukemias (CML, ALL), fibrosarcoma, myelodysplastic/proliferative disorders, systemic mastocytosis	Congestive heart failure, tachycardia, palpitations, pulmonary and peripheral edema, arrhythmia, atrial fibrillation, cardiac arrest, myocardial infarction, angina pectoris, pericardial effusion, hypereosinophilic cardiotoxicity. Vascular: flushing, hemorrhage, hyper- or hypotension, Raynaud’s phenomena, thromboembolism. Elevations in CPK and LDH.
RA receptor agonists	Bexarotene (Targretin)	retinoid	Cutaneous T cell lymphoma	Lipid effects (increased cholesterol, triglycerides & LDL, decreased HDL) in up to 75% of patients. Edema, hyperglycemia, hypothyroidism, hypertension, angina pectoris, right heart failure, syncope, and tachycardia (infrequent, but reported).
	Alitretinoin	retinoid	Cutaneous Kaposi’s sarcoma	Topical gel, limited systemic effects, can produce
	(Panretin)		(AIDs-related)	edema
	Tretinoin (Aberel)	retinoid	Acute promyelocytic leukiemia (APL)	Retinoid acid-APL syndrome (up to 25% of APL patients, see text) including pericardial and pleural effusions, depressed LV function and hypotension. Hemorrhage (including cerebral), chest pain, flushing, edema (including peripheral and pulmonary), arrhythmia (including cardiac arrest), hyper- or hypotension, MI, pericarditis, pulmonary hypertension, LV dilation, murmur, cardiomyopathy, thromboembolism, pseudotumor cerebri [147]. Hypertriglyceridemia, hypercholesterolemia.
Proteosome inhibitors	Bortezomib (Velcade)	SMI	Multiple myeloma, mantle cell lymphoma	Hypotension, exacerbation of or new onset heart failure, decreased LV systolic function, QT prolongation, pulmonary hypertension, hyper- or hypoglycemia
Abl and Src families, c-Kit, EphA2,	Dasatinib (Sprycel)	SMI	Leukemias (CML, ALL)	Vascular effects (flushing, hypertension, thrombophebitis, thrombosis/embolism), QT prolongation, atrial arrhythmias, congestive heart
and PDGFRβ.				failure.
Abl, c-Kit, PDGFR, CSF-1R, DDR1	Nilotinib (Tasigna)	SMI	CML	Vascular effects (hypertension), QT prolongation, angina, arrhythmia (sudden death, atrial fibrillation, AV block and others).
**mTOR**				Vascular endothelium, cardiac myocyte
	Temsirolimus (Torisel)	SMI	Renal cell carcinoma	Vascular (hypertension, thromboembolism, thrombophebitis), chest pain, increases in serum cholesterol, triglycerides and glucose.
	Everolimus (Afinitor)	SMI	Renal cell carcinoma, subependymal giant cell astrocytoma with tuberous sclerosis	Chest pain/jaw pain, hypertension, tachycardia, congestive heart failure increases in serum cholesterol, triglycerides and glucose, (limited study showed no QT prolongation [141]).
**Hormone Signaling Pathways**				Vascular endothelium, cardiac myocytes
Estrogen Receptor (SERMs)	Tamoxifen (Nolvadex)	Non-steroidal anti-estrogen	ER+ breast cancers	Vascular (thromboembolism, stroke, vasodilation, hypertension), peripheral edema, angina & myocardial infarction (may be decreased [142, 143]), lipid effects (decreased cholesterol and LDL) may be beneficial [108].
	Toremifene (Fareston)	Non-steroidal anti-estrogen	ER+ breast cancers	Cardiac Failure, myocardial infarction, arrhythmia, angina pectoris [144], thromboembolism, stroke/TIA, lipid effects (decreased cholesterol, LDL and increased HDL [107, 108]).
	Fulvestrant (Faslodex)	ER antagonist	ER+ breast cancers	Vascular (vasodilation, hot flashes), peripheral edema.
Aromatase inhibitors	Anastrozole (Arimidex)	Non-steroidal inhibitor	ER+ breast cancers	Vascular (vasodilation, hot flashes; thrombophebitis), hypertension, peripheral edema, myocardial ischemia (risk may be increased in patients with underlying CAD), cholesterol increased (compared to tamoxifen [105]).
	Exemestane (Aromasin)	Steroidal inhibitor	ER+ breast cancers	Vascular (vasodilation, hot flashes), hypertension.
	Letrozole (Femara)	Non-steroidal inhibitor	ER+ breast cancers	Vascular (vasodilation, hot flashes), edema, cholesterol increased (at 24 months in phase III trial, but not at 5 years), hypertension, chest pain (not significantly higher than seen with tamoxifen), thromboembolism. Cardiovascular (ischemic, CAD, palpitations, tachycardia) & cerebrovascular (stroke, TIA) events, <2% [145].
**Regulators of Gene Expression, Cell Survival and Other Functions**				All cell types
HDAC inhibitors	Vorinostat (Zolinza)	SMI	Cutaneous T cell lymphoma	Thromboembolism, myocardial infarction & stroke (single reports), chest pain, hyperglycemia, QT prolongation [146].
	Romidepsin (Istodax)	SMI	Cutaneous T cell lymphoma	Arrhythmia (SVT, VT), ST-T wave changes, QT prolongation, edema, hyperglycemia
RA receptor agonists	Bexarotene (Targretin)	retinoid	Cutaneous T cell lymphoma	Lipid effects (increased cholesterol, triglycerides & LDL, decreased HDL) in up to 75% of patients. Edema, hyperglycemia, hypothyroidism, hypertension, angina pectoris, right heart failure, syncope, and tachycardia (infrequent, but reported).
	Alitretinoin	retinoid	Cutaneous Kaposi’s sarcoma	Topical gel, limited systemic effects, can produce
	(Panretin)		(AIDs-related)	edema
	Tretinoin (Aberel)	retinoid	Acute promyelocytic leukiemia (APL)	Retinoid acid-APL syndrome (up to 25% of APL patients, see text) including pericardial and pleural effusions, depressed LV function and hypotension. Hemorrhage (including cerebral), chest pain, flushing, edema (including peripheral and pulmonary), arrhythmia (including cardiac arrest), hyper- or hypotension, MI, pericarditis, pulmonary hypertension, LV dilation, murmur, cardiomyopathy, thromboembolism, pseudotumor cerebri [147]. Hypertriglyceridemia, hypercholesterolemia.
Proteosome inhibitors	Bortezomib (Velcade)	SMI	Multiple myeloma, mantle cell lymphoma	Hypotension, exacerbation of or new onset heart failure, decreased LV systolic function, QT prolongation, pulmonary hypertension, hyper- or hypoglycemia

* Information extracted from data package inserts and other information from manufacturers (available at the FDA website: http://www.fda.gov/) unless otherwise noted. Thus, for many reported side effects, there is limited data re. frequency in larger populations.

## ABBREVIATIONS USED (INCLUDING TABLE)

Abl: = Abl kinase, fused with Bcr in CML
ALL: = Acute lymphoblastic leukemia
AV: = Atrioventricular
CAD: = Coronary artery disease
CML: = Chronic myelogenous leukemia
CNS: = Central nervous system
CSF-1R: = Colony stimulating factor-1 receptor
DDR1a: = Discoidin domain receptor tyrosine kinase 1
EGFR: = Epidermal growth factor receptor
EphA2: = Ephrin A2 receptor
ER (deg): = Promote targeted degradation of ER
FLT3: = Fms-like tyrosine kinase-3
GE: = Gastroesophageal
GI: = Gastrointestinal
HDAC: = Histone deacetylase
Kit: = c-Kit (CD117) kinase, receptor for stem cell factor
LV: = Left ventricle
LVEF: = LV ejection fraction
mAb: = Monoclonal antibody
MI: = Myocardial infarction
NSCLC: = Non small cell lung cancer
PDGFR: = Platelet derived growth factor receptor
Raf: = An intracellular signaling serine/threonine kinase
RAR: = Retinoic acid receptor
RET: = Glial cell-line derived neurotrophic factor receptor
SERMs: = Selective ER modulators, bind to hormone binding domain to modulate ER function
SMI: = Small molecule inhibitor
SVT: = Supraventricular tachycardia
TIA: = Transient ischemic attack
TK: = Tyrosine kinase
VEGF: = Vascular endothelial growth factor
VEGFR: = VEGF receptor
VT: = Ventricular tachycardia
